# Jujube powder supplementation optimized high-moisture alfalfa silage through regulating microbial community

**DOI:** 10.3389/fmicb.2025.1740083

**Published:** 2026-01-12

**Authors:** Zhenyu Liu, Pengfei Liu, Nan Xie, Jianfei Zhi, Haoyu Wang, Shenglin Hou, Xuewei Pang, Entao Wang, Guoli Hu, Lige Guo, Zhongkuan Liu, Xiaoyun Liu

**Affiliations:** 1 Hebei Key Laboratory of Soil Fertilization and Agricultural Green Development, Hebei Fertilizer Technology Innovation Center, Institute of Agro-Resources and Environment, Hebei Academy of Agriculture and Forestry Sciences, Shijiazhuang, China; 2 Engineering Research Center of Ecological Safety and Conservation in Beijing-Tianjin-Hebei (Xiong’an New Area) of MOE, Key Laboratory of Microbial Diversity Research and Application of Hebei Province, Engineering Laboratory of Microbial Breeding and Preservation of Hebei Province, College of Life Science, Hebei University, Baoding, China; 3 Departamento de Microbiología, Escuela Nacional de Ciencias Biológicas, Instituto Politecnico Nacional, Mexico, DF, Mexico

**Keywords:** alfalfa silage, bacterial community, fermentation, functionality, jujube powder

## Abstract

In order to improve the quality of fresh alfalfa silage, we investigated the effects of jujube powder (JP) addition on the ensiling process of high-moisture alfalfa (75% moisture content) over fermented periods of 1, 5, 15, 30, 45 and 60 days. We evaluated chemical composition, bacterial and fungal community dynamics and diversity, co-occurrence networks, microbial functionality and the ability to restrict pathogenic contamination. Results showed that JP addition optimized key fermentation parameters (pH, lactic acid, volatile fatty acids, and NH_3_-N) in high-moisture alfalfa, achieving levels comparable to those of traditional wilted alfalfa qualified silage. JP also increased the abundance of beneficial bacteria while suppressing undesirable organisms. A dominant lactic acid bacteria (LAB) combination—*Lactobacillus-Pediococcus-Lactococcus* was observed, showing strong positive correlations with silage quality indicators (higher qualified LAB counts and NH_3_-N content, and lower butyric and propionic acid levels). Metabolic pathway analysis revealed that JP supplementation effectively restricted the amino acid metabolism in harmful bacteria while significantly enhancing key carbohydrate-utilization pathways. Notably, D-alanine was unregulated in JP-treated, supporting the survival and function of LAB as the primary fermentation agents. In contrast, the biosynthesis and degradation of branched-amino acids (valine, leucine and isoleucine) remained stable, contributing to better protein preservationl. Furthermore, JP addition helped control plant and animal pathogens and limited saprotrophic activity. In conclusion, by adding JP, the energy- and labor-intensive pre-wilting procedure could be replaced for optimizing the high-moisture alfalfa silage. This work also identifies *Lactobacillus-Pediococcus-Lactococcus* as a promising microbial combination for future inoculant development.

## Graphical abstract

**Figure:**
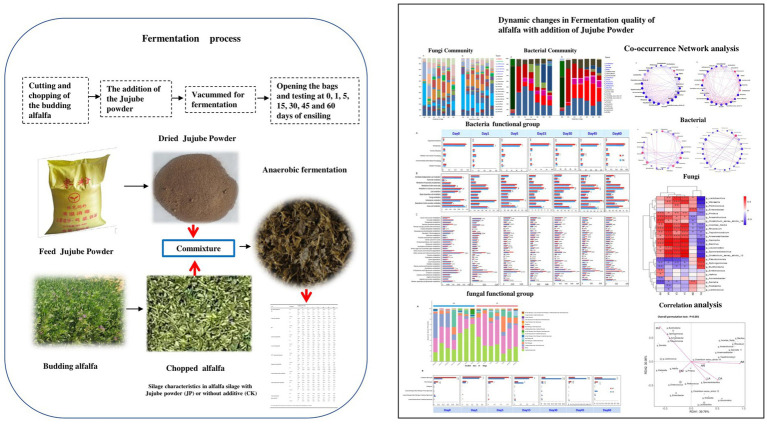

## 1 Introduction

Ensiling is an effective technique for preserving high-quality forage from green plants (e.g., forage corn), agricultural by-products (e.g., corn stalks, wheat straw, sweet potato vines), and forage crops (e.g., alfalfa) to address winter and early spring feed shortages in northern China. During ensiling, water-soluble carbohydrates (WSCs) are mainly converted into lactic and acetic acids through anaerobic fermentation. This traditional technology could enhance feed palatability and extends storage time of green forages (Ren et al., 2020). As one of the most important foliage crops, alfalfa (*Medicago sativa* L.) is also an excellent ensiling material due to its extensive cultivation, high yield, and superior digestibility (Dunière et al., 2013; Kung et al., 2018; Oliveira et al., 2017). In China, alfalfa cultivation covered approximately 5.5 × 10^5^ ha with a production of 5.0 × 10^6^ tons in 2023.^1^ As a legume, alfalfa possesses a deep root system with nitrogen fixing nodule symbiosis with rhizobia (*Sinorhizobium meliloti*) (Wang et al., 2018). Its high protein, vitamin, mineral, and fiber contents make it an excellent dietary component for livestock, particularly for dairy cows to improve the milk production (Albrecht and Beauchemin, 2003; Muck et al., 2018). Although alfalfa hay can be stored to mitigate winter feed shortages, its silage can preserve more nutrients by reducing leaf loss.

Silage quality and nutrient preservation depend on multiple factors, including crop species, ensiling technologies, machinery and additive (Kung et al., 2018). Despite its advantages, alfalfa is challenging to ensile due to its high buffering capacity from the high protein content, low WSC content (<1.5%), and often insufficient dry matter (<30%) in the raw forage. These characteristics promote secondary fermentation by *Clostridia* to convert lactic acid into butyric acid, which leads to pH increases and spoilage of the silage. To address these limitations, strategies including wilting to reduce moisture, mixing with dry ingredients, or applying additives of carbohydrate-rich organic materials alone or combined with lactic acid bacteria (LAB) inoculation have been used to inhibit aerobic bacteria and improving silage quality (Kung et al., 2018). It has been reported that both chemical (e.g., sugars and organic acid) and biological (e.g., LAB, enzymes) additives could prolong the preservation time, increase the protein retention, and regulate the carbohydrate degradation by suppressing Clostridia and other detrimental microbes (Hu et al., 2021; Ni et al., 2017; Queiroz et al., 2018; Wang et al., 2020).

Compared with chemical additives, biological additives, including high C/N ratio plants and agricultural by-products, are more sustainable due to their low cost and eco-friendliness properties (Luo et al., 2021; Tatlı et al., 2024). One promising candidate is jujube powder, a by-product of Chinese date (*Ziziphus jujuba* Mill.) production. Tian et al. (2017) demonstrated that jujube powder could improve alfalfa silage fermentation by enhancing nutritional quality and nitrogen fractions, especially when combined with LAB inoculation. *Ziziphus jujuba* belongs to the *Rhamnaceae* family, and preserves in two main varieties, the shrub-type wild variety (*Z. jujuba* var. spinose) for producing sour jujube and tree-type cultivated variety (*Z. jujuba* var. *jujuba*) widely yields grown in northern China for producing jujube (Chinese date) (Shao et al., 2024). Beyond its use as a nutrient-rich food, flavoring agent, and herbal medicine (Ruan et al., 2024), jujube has been increasingly processed into livestock feed in the form of powder, often made from low-grade fruits, waste pulp, or residues blended with rice husk (Li et al., 2009). This powder is rich in fermentable sugars, amino acids, vitamins, minerals and epiphytic microorganisms. Previous studies have evidenced that jujube powder supplementation could reduce moisture content, decrease buffering capacity, promote LAB growth, accelerate pH decline, and minimize water-soluble carbohydrate (WSC) loss (Tian et al., 2017; Liu et al., 2016; Weiss and Underwood, 2009), and also suppress ammonia-N accumulation, clostridial activity, and mold development (Rajabi et al., 2017). However, it remains unclear whether jujube powder alone can enable high-moisture alfalfa silage to achieve quality comparable to traditionally wilted silage, thereby eliminating the need for pre-wilting. Moreover, how jujube powder systematically regulates microbial community succession, interactions, and functional dynamics during ensiling has not been thoroughly investigated.

To clarify these gaps, this study evaluate the effects of jujube powder supplementation on high-moisture alfalfa silage under vacuum fermentation conditions, with the aim of assessing its potential to replace energy- and labor-intensive wilting practices while elucidating its modulatory effects on the silage microbial ecosystem.

## 2 Materials and methods

### 2.1 Silage preparation and sampling

Second-cut alfalfa (*Medicago sativa* L.) at early bud stage was harvested from an experimental field (117°49′E, 39°39’N) in Huanghua City, Hebei Province, China. The Crop was grown without herbicide or fertilizer application. Fresh alfalfa was chopped to 2–3 cm in length. Jujube powder was obtained from Cangzhou Defeng Jujube Industry Co., Ltd. (Qingtown of Cangzhou City, Hebei Province). Two treatments were prepared: (i) control (CK) composed of 300 g chopped alfalfa; and (ii) Jujube powder treatment composed of 300 g chopped alfalfa supplied with 4% (w/w) Jujube powder according to a previous study (Liu et al., 2016). Chemical characters and microbial compositions of the raw materials are shown in Table 1. The mixed materials were packed manually into 28 cm × 35 cm polyethylene bags and vacuumed (DZ-360 vacuum sealer. Jinqrui Co. Wuxue City, Hubei, China). A total of 42 bags (2 groups × 3 replicates × 7 sampling times) were prepared and stored at ambient (25–37 °C). Sampling was conducted at 0, 1, 5, 15, 30, 45 and 60 days of ensiling in triplicate. For each bag, 10 g of silage was collected, homogenized, and stored at −80 °C for microbial analysis. The remaining material was used for fermentation parameter analysis.

**Table 1:** Chemical characters and microbial compositions of raw materials used for ensiling.

Characters	Feed jujube powder	SD	Alfalfa in early bloom	SD	*p*-value of JP vs. alfalfa
pH	5.20	0.12	6.42	0.14	0.001
Dry matter (g/kg FW)	93.28	3.78	25.31	1.75	0.001
Water soluble carbohydrate (g/kg DM)	30.23	1.34	5.96	0.06	0.001
Crude protein (g/kg DM)	6.55	0.67	23.32	2.30	0.001
Neutral detergent fiber (g/kgDM)	34.15	2.34	37.43	3.78	0.453
Acid detergent fiber (g/kg DM)	25.18	1.97	32.18	2.89	0.500
Ammonia-N	ND		ND		
Organic acids	ND		ND		
Lactic acid bacteria (Log_10_ CFU/g FW)	6.38	0.05	5.15	0.34	0.001
Yeast (Log_10_ CFU/g FW)	0		3		0001
Moulds (Log_10_ CFU/g FW)	3.0	0.21	4.54	0.56	0.001
Aerobic bacteria (Log_10_ CFU/g FW)	4.42	0.33	4.92	0.43	0.001

### 2.2 Chemical composition and fermentation analysis

Chemical composition of the samples was analyzed according to standard methods. Dry matter content was determined by the weigh difference before and after drying the sample at 65 °C for 48 h. The dried sample was ground through 1.0 mm sieve for the subsequent nutrient analysis. Crude protein was analyzed according to standard procedure detailed by the Association of Official Analytical Chemists (Hasan, 2015). Neutral detergent fiber (NDF) and acid detergent fiber (ADF) were measured according to the method of Van Soest et al. (1991) by using fiber analyzer (Ankom 2000i full; Ankom Tech Co., Macedon, NY, United States).

To determine the fermentation parameters, fresh silage sample (25 g × 3) was mixed with 225 mL sterile water and incubated at 4 °C overnight, then homogenized for 1 min and filtrated with quantitative filter paper. The acquired filtrate was centrifuged at 4 °C, 4500 × g, for 15 min and the obtained supernatant was conducted for measuring pH with a pH meter (Mettler Toledo). Lactic, acetic, propionic, and butyric acids were quantitively estimated by HPLC (Shimadzu, Tokyo, Japan) equipped with a UV detector and set as follows: Shodex Rspak KC-811S-DVB gel column, eluent 3 mmol/L perchloric acid at a running rate of 1.0 mL/min, temperature of column oven 50 °C; wavelength of 210 nm, injection volume of 5 μL. NH_3_-N content was measured by phenol-sodium hypochlorite method (Broderick and Kang, 1980). Water-soluble carbohydrate content was determined by the anthrone-sulfuric acid colorimetric method (McDonald and Henderson, 1964).

### 2.3 Quantification of culturable microbes and analysis of microbial community composition associated with jujube powder and alfalfa

For counting the culturable lactic acid bacteria, yeasts and molds, each of the samples (20 g) was immediately blended with 180 mL sterilized saline solution (NaCl 8.5 g/L), and serially diluted. Aliquots (0.1 mL) of the dilutions were spread on plates of De Man–Rogosa–Sharpe agar (MRS) (1.10660, Millipore, according to ISO 15214, for Lactobacilli) and Rose Bengal agar (R1273, Millipore, for yeasts and fungi). The colony forming units were counted after incubation of 2–5 days at 28 °C (Wang et al., 2017).

The samples frozen at −80 °C were used for metagenomic DNA extraction according to Liu et al. (2019). Three repeat samples (10 g × 3) for each day and total 42 samples were respective mixed with 90 mL of sterile normal solution with vigorous shaking at 120 r/m for 2 h. Then the mixture was filtered and the filtrate was centrifuged at 10,000 rpm for 10 min at 4 °C. The deposit was suspended in 1 mL of sterile saline solution and the microbial pellets for DNA extracting were obtained by centrifugation at 12,000 rpm for 10 min at 4 °C. Then DNA was extracted using the MN NucleoSpin 96 Soi (Macherey Nagel, Düren, GA, United States) according to the manufacture’s protocols. All the metagenomic DNA extracts were sent to Beijing Baimaike Biotechnology Co., Ltd. (Beijing, China) for microbial community estimation through the paired-end high-throughput sequencing with Illumina HiSeq 2500 platform. For bacteria, the 16S rDNA V3–V4 variable region was amplified by PCR using the universal primers with barcode: 338F (5′-ACTCCTACGGGAGGCAGCA-3′) and 806R (5′-GGACTACHVGGTATCTAAT-3′) (Ding et al., 2020). For fungi (including yeasts), the ITS region was amplified with the primers ITS1F (5′-CTTGGTCATTTAGAGGAAGTAA-3′) and ITS2 (5′-GCTGCGTTCTTCATCGATGC-3′) (De Beeck et al., 2014). Three technical repetitions were sequenced for each sample. The reads in the range of 480–490 bp was retained after quality control filtering and the effective sequences were clustered into operational taxonomic units (OTUs) at the threshold of 97% similarity using Uparse pipeline (version 7.0, Edgar, 2013) based on the database of Silva 138 and Unite 7.0 (Quast et al., 2013; Pruesse et al., 2007).

ACE and Shannon index of alpha-diversity were evaluated with Mothur software (version 7.0, Schloss et al., 2009). The principal coordinate analysis (PCoA) was performed by R software (5.2) to assess community dissimilarity among samples, using Bray–Curtis distance. The co-occurrence patterns among bacterial and fungal community and redundancy analysis were analyzed in cloud platform of https://www.bioincloud.tech/. LefSe was conducted by Metastats to explore the dynamic change of microbial community during ensiling. Microbial function prediction was estimated at the Kyoto Encyclopedia of Genes and Genomes (KEGG) database using Phylogenetic Investigation of Communities by Reconstruction of Unobserved States (PICRUSt2) (Douglas et al., 2020) and FUNGuild (Nguyen et al., 2016).

### 2.4 Statistical analysis

Chemical composition and microbial community data were compared by one-way ANOVA with student test (*p* < 0.05) by SPSS 18.0 (IBM, United States). The α-diversity indices and β-diversity were estimated by permutation test, while PERMANOVA was used to analyze the PCoA and RDA results.

## 3 Results

### 3.1 Properties of jujube powder and fresh alfalfa

The chemical and microbial compositions of jujube powder and fresh alfalfa are shown in Table 1. Fresh alfalfa showed typical characteristics with pH 6.42, 25.31% dry matter, and 5.96% water-soluble carbohydrates, while containing no detectable organic acids or NH_3_-N. In contrast, jujube powder exhibited significantly different properties with lower pH (5.20), higher dry matter (93.28%), and substantially greater water-soluble carbohydrate content (30.23%), but lower crude protein (6.55%) in comparison with alfalfa. Meanwhile, their neutral detergent fiber (NDF) and acid detergent fiber (ADF) proportions were comparable. Furthermore, JP contained a substantially more lactic acid bacteria (LAB) and less aerobic bacteria/molds than alfalfa did (Table 1).

### 3.2 Dynamics of alfalfa ensiling with/without JP addition

The fermentation dynamics and chemical compositions of alfalfa silages with or without JP addition are presented in Table 2. In both the treatment groups, pH was dropped rapidly on the first day of fermentation. However, JP addition significantly (*p* < 0.005) reduced initial pH and maintained lower pH levels throughout the ensiling compared to the control (CK), though a slight pH increase occurred on day 5 in both groups, possibly due to reduced organic acid production and NH_3_-N release from protein degradation on this time. During the whole fermentation procedure, pH value was decreased continuously alongside increasing organic acid accumulation, particularly lactic acid (LA). While pH in CK stabilized at 4.8 by day 30, it further decreased in JP group, reaching 4.26 by day 60 (*p* < 0.005).

**Table 2:** Silage characteristics in alfalfa silage with jujube powder (JP) or without additive (CK).

Items	Treatment	Sampling time (day)							
			0	1	5	15	30	45	60
Dry matter (g/kg FW)	CK	Mean	221.3	218.7	218.4	216	224.6	207.2	207.8
SD		SD	2.2	6.1	5.8	4.6	9.1	2.2	2.6
	JP	Mean	258.1	258.9	252.2	273.4	263.4	252.2	257.8
		SD	1.3	1.6	4.2	6.5	8.5	4.3	5.8
	CK × JP	P	<0.001	<0.001	0.006	0.002	0.024	0.002	0.001
Crude ash (g/kg DM)	CK	Mean	123.66	123.88	138.24	147.56	149.18	149.12	152.40
		SD	1.95	1.85	4.3	5.6	2.1	1.5	3.7
	JP	Mean	141.13	127.16	142.79	152.96	151.54	151.46	151.31
		SD	2.3	0.6	2.3	3.6	3.6	4.3	5.5
	CK × JP	P	0.007	0.555	0.119	0.087	0.447	0.403	0.712
pH	CK	Mean	6.42	6.15	5.49	5.38	4.84	4.86	4.79
		SD	0.032	0.055	0.1	0.035	0.1	0.11	0.25
	JP	Mean	5.85	5.55	4.82	4.52	4.45	4.36	4.26
		SD	0.092	0.131	0.11	0.04	0.029	0.05	0.06
	CK × JP	P	<0.001	0.002	0.002	<0.001	0.005	0.002	0.003
Lactic acid (g/kg DM)	CK	Mean	8.7	10.70	13.20	17.30	36.90	34.10	32.70
		SD	0.05	0.04	0.73	0.9	0.43	0.84	0.72
	JP	Mean	16.40	17.12	17.90	19.40	44.50	41.30	35.60
		SD	0.05	0.06	0.04	0.7	1.3	2.7	5.12
	CK × JP	P	0.021	0.119	0.022	0.492	0.046	0.008	0.006
Acetic acid (g/kg DM)	CK	Mean	2.20	2.30	2.00	2.00	5.50	5.30	8.00
		SD	0.06	0.73	0.56	0.85	0.84	0.56	0.61
	JP	Mean	1.90	2.20	2.50	2.90	3.40	4.10	5.60
		SD	0.36	0.01	0.19	0.55	0.13	0.47	0.51
	CK × JP	P	0.001	0.129	0.002	0.021	0.006	0.078	0.002
NH_3_-N (g/kg total nitrogen)	CK	Mean	78.30	154.59	275.35	546.77	538.56	433.41	384.31
		SD	4	10.4	8.4	10.4	8.5	4.4	3.5
	JP	Mean	44.9	67.37	143.57	163.38	231.9	153.5	151.8
		SD	1.05	2.1	4.7	8.1	5.7	7.1	5
	CK × JP	P	5.19	0.02	1.34	0.49	0.09	1.57	0.04
Propionic acid (g/kg DM)	CK	Mean	0.07	0.07	0.07	0.37	2.80	4.00	5.00
		SD	0	0	0	0.05	0.81	0.36	0.56
	JP	Mean	0.057	0.056	0.058	0.050	0.982	1.800	2.100
		SD	0.000	0.000	0.000	0.000	0.300	0.500	0.600
	CK × JP	P					<0.001	<0.001	<0.001
Neutral detergent fiber (g/kg DM)	CK	Mean	289.9	362.4	359.6	298.1	332.1	345.3	306.9
		SD	6.35	83.2	52.3	6.53	35.1	5.3	45.2
	JP	Mean	306.8	326.2	365.3	325.1	352.7	342.6	321.6
		SD	21.3	21.3	26.3	24.3	25.9	34.2	2.31
	CK × JP	P	0.052	0.032	0.021	0.03	0.032	0.32	0.032
Acid detergent fiber (g/kg DM)	CK	Mean	299.9	289.2	296.6	392.5	298.3	321.6	311.2
		SD	23.1	25.1	9.65	45.32	23.1	12.3	15.6
	JP	Mean	281.5	273.9	285.6	293.7	290.1	293.1	301.2
		SD	21.9	12.3	15.8	35.6	29.1	35.2	12.3
	CK × JP	P	0.057	0.098	0.026	0.054	0.32	0.54	0.6
Lactic acid bacteria (Log_10_ CFU/g FW)	CK	Mean	4.301	7.279	8.398	8.977	7.204	7.041	6.919
		SD	0.23	0.45	0.12	0.987	0.34	0.67	0.87
	JP	Mean	5.23	7.522	9.077	9.544	7.53	7.114	6.806
		SD	0.12	0.76	0.34	0.87	00.67	0.76	0.78
	CK × JP	P	0.003	0.005	0.001	0.005	0.045	0.07	0.04

FW, fresh weight; DM, dry matter; ND, not detected; CFU, colony-forming units; SD, standard error of means.

The organic acid profiling revealed LA, acetic acid (AA), and propionic acid (PA) as dominant fermentation products, with no detectable butyric acid (BA) by the end of fermentation in both the silages with/without JP addition. In the final silage (60 days of fermentation), JP-treated silage vs. CK presented greater DM content (257.8 vs. 207.8%, *p* = 0.001) and LA content (35.6 vs. 32.7, *p* < 0.01); but lower pH (4.3 vs. 4.8, *p* < 0.005), acetic acid content (6.6 vs. 8, *p* < 0.005) and propionic acid content (2.1 vs. 5, *p* < 0.001).

Correspondingly, the LA:AA ratio in CK ranged between 4.09 (day 60) and 8.65 (day 15), whereas JP-treated silage exhibited higher ratios (6.52–13.09), indicating enhanced homolactic fermentation. As shown in Table 2, AA levels increased similarly in both groups during the first 15 days but were significantly lower (*p* < 0.01) in JP from day 30 onward, this decrease coinciding with a slight LA decline and continued accumulation of AA. PA followed a trend similar to AA, with JP significantly reducing PA concentrations in the later stages.

NH_3_-N levels, which reflecting protein degradation, remained below the recommended threshold (<150 g/kg total N) in both treatments. However, JP addition significantly (*p* < 0.01) reduced NH_3_-N compared to CK. Dry matter content was consistently higher in JP-treated silage (around 260 g/kg FW) than in CK (around 210 g/kg FW) throughout the ensiling process, matching pre-ensiling measurements. In contrast, ash content remained unaffected by JP treatment.

### 3.3 Cultural *Lactobacillus* bacteria population in alfalfa ensiling process

JP treatment significantly enhanced the population of culturable lactic acid bacteria (LAB) only at the initial stage of ensiling (Table 2), consistent with the 12.5-fold higher LAB content in the Jujube Powder compared to the raw alfalfa (Table 1). Both JP-treated and control (CK) silages showed similar dynamic patterns of LAB: a rapid increase on day 1, peaking at day 15, followed by gradual decline until fermentation completion, with no significant differences between treatments (Table 2). The LAB abundance dynamics displayed an inverse relationship with some key fermentation parameters: when the LAB abundance increased rapidly, the concentrations of lactic acid, acetic acid and propionic acid changed minimally or remained stable during the initial 15-days; and when the LAB population decline, the concentrations of lactic acid, acetic acid and propionic acid content increased during the subsequent 15–60 days (Table 2).

### 3.4 Diversity and composition of microbial community in alfalfa silage

**Figure 1:**
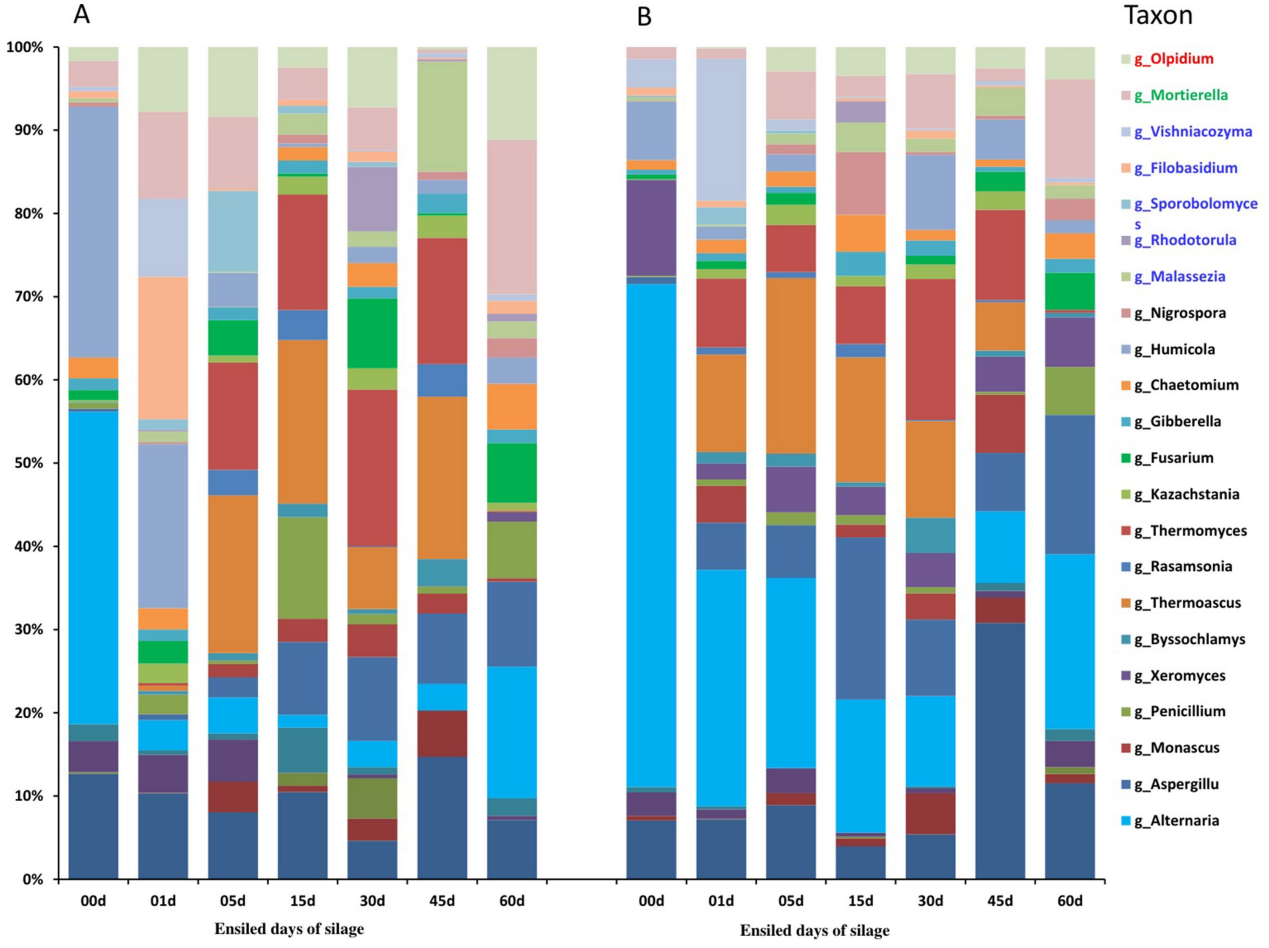
Bar plots showing the dynamic changes of the top fungi OTUs in alfalfa silage based upon the metagenomic analysis. **(A)** Control. **(B)** Alfalfa with 4% of jujube powder, and the blue letter of taxon belonging to Basidiomycota, while the black letter of taxon belonging to Ascomycota, and green letter of taxon belonging to Mortierellomycotaes, the red letter of taxon belonging to Olpidiomycota.

The microbial community analysis with high throughput sequencing revealed distinct patterns in alfalfa silage with and without JP addition. From the 42 DNA samples (in 3 replications), a total of 4,039,918 raw pair reads of fungal ITS were obtained and checked using FLASH (version 1), and 2,725,262 clean tags were obtained by two sections splicing. Fungi presented in relatively low abundances throughout the ensiling process in both the treatments (Figure 1; Supplementary Figure S2 and Supplementary Tables S1, S2). At genus level, 27 genera with more than 1% relatively abundances (Supplementary Table S2) were detected, with *Humicola* and *Alternaria* being the most dominant; in JP-treated silage, *Alternaria* declined by ensiling from 60.4% in raw material, and reached the lowest peak (8.6%) on day 45 (Figure 1 and Supplementary Table S2). Importantly, JP addition significantly reduced populations of several molds and yeasts including *Humicola*, *Thermoascus*, *Thermomyces*, *Cladosporiuma*, *Aspergillus*, *Penicillium*, *Fusarium* and *Olpidium* compared to the control.

High-throughput sequencing of 16S rRNA genes generated 6,775,441 quality-filtered 16S rRNA gene sequences, with mean coverage exceeding 4×, ensuring reliable results. From the 42 DNA samples (in 3 replications), a total of 7,522,867 raw pair reads were obtained and checked using FLASH (version 1), and 6,775,441 clean tags were obtained by two sections splicing. Shannon diversity indices increased progressively during ensiling (0.46 to 0.78 in CK; 0.63 to 0.91 in JP), except for day 1 in JP treatment (Supplementary Figure S1A and Supplementary Table S3). JP-treated ensiling showed significantly higher (*p* < 0.001) Shannon indices than CK at most the time points, likely due to initial community differences caused by JP addition. ACE, and Chao1 indices indicated greater initial richness in JP treatments and they were decreased during ensiling (Supplementary Figures S1B–D), while Simpson indices mirrored Shannon diversity patterns (Supplementary Figure S1C).

**Figure 2:**
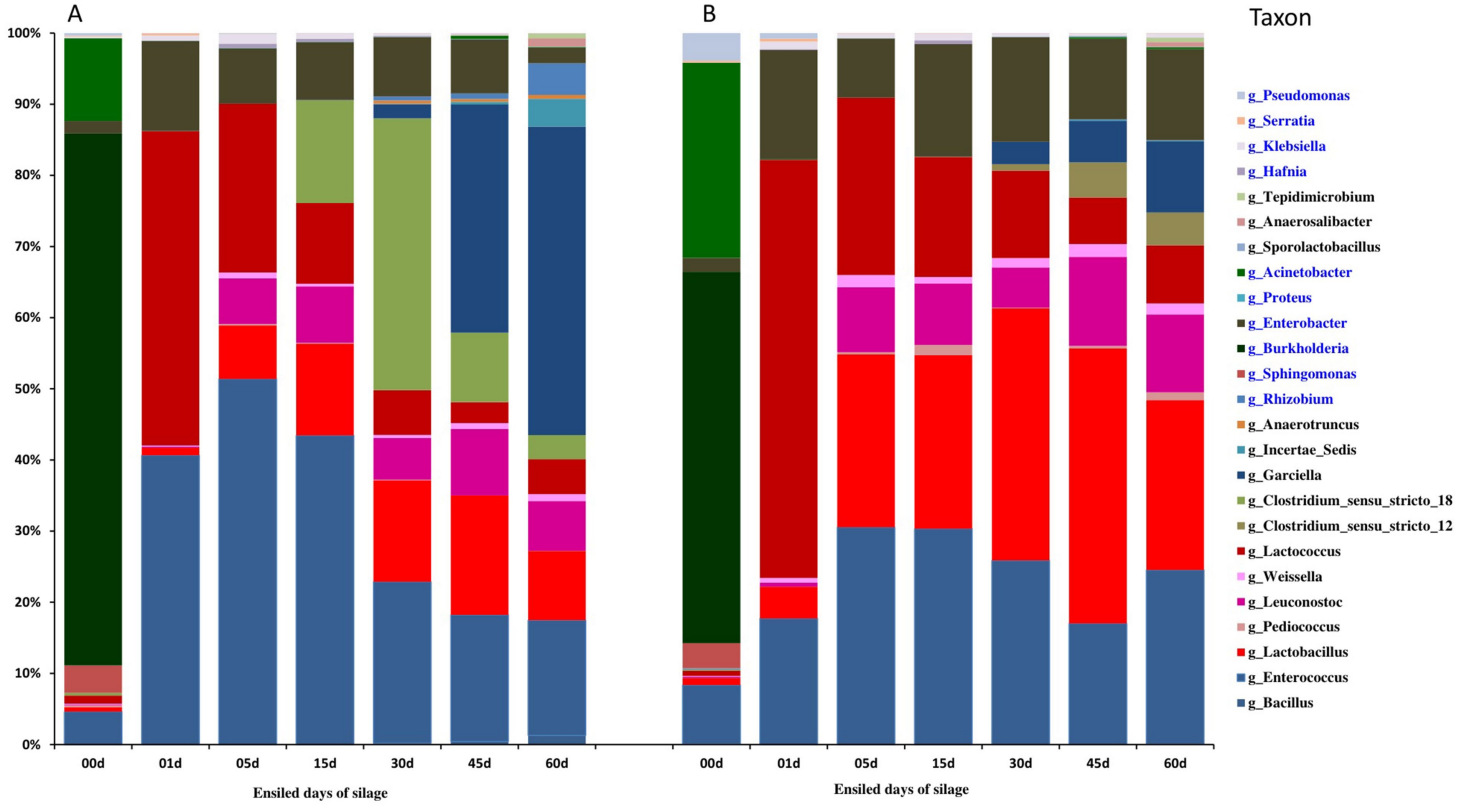
Bar plots showing the dynamic changes of the top 25 bacterial OTUs in alfalfa silage based upon the metagenomic analysis. **(A)** Control. **(B)** Alfalfa with 4% of jujube powder, and the blue letter of taxon belonging to Proteobacteria, while the black letter of taxon belonging to Firmicutes.

**Figure 3:**
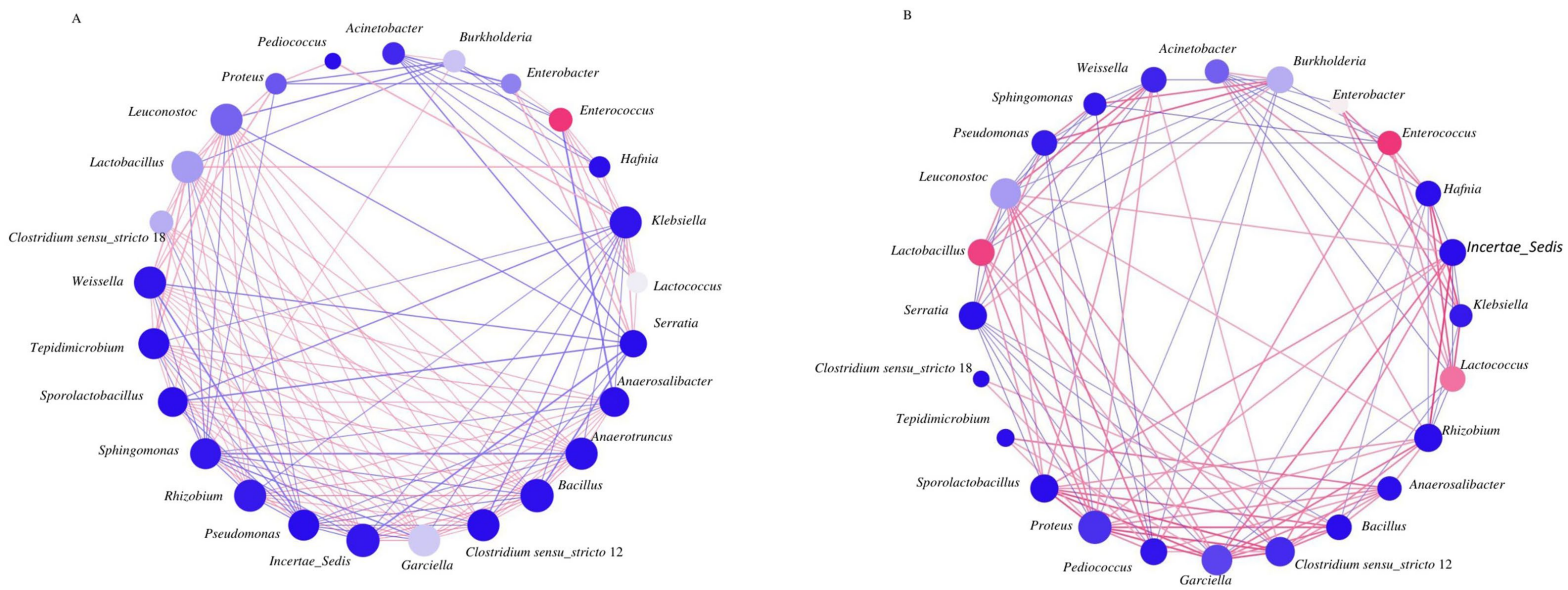
Co-occurrence network analysis among bacterial OTUs (species) with relative abundances >1% in alfalfa silage for 60 days of fermentation. Bacterial co-occurrence networks (Spearman correlation, the most abundant 25 species, *p*-value <0.05, correlation >0.5) of Control **(A)**, alfalfa with 4% of jujube powder **(B)**. The node represents bacterial species, node color represents bacterial abundance, and node size represents the degree. Edges are colored according to positive (red) and negative (blue) correlations.

Analysis of the top 25 operational bacterial taxonomic units (OTUs) revealed 17 families containing 25 genera and 5 defined species (Figure 2 and Supplementary Tables S1, S4). JP addition initially increased abundances of *Lactobacillus*, *Enterococcus* and *Lactococcus*, while decreased those of *Burkholderia*, *Sphingomonas* and *Pediococcus pentusaceus* compared to CK (Figures 2, 3 and Supplementary Table S4).

Both treatments showed rapid disappearance of *Burkholderia*, *Acinetobacter* and *Sphingomonas* by day 1, coinciding with pH drop and LAB increase (Figures 2, 3; Supplementary Figure S2 and Table 2), suggesting oxygen consumption by the aerobic bacteria facilitated anaerobic LAB growth. Subsequent fermentation demonstrated similar successional patterns for dominant OTUs (*Enterococcus*, *Lactococcus*, *Lactobacillus*, *Leuconostoc*); timing differed *Lactococcus* peaked at day 1 then declined, while *Enterococcus*, *Lactobacillus* and *Anaerosalibacter* peaked on days 5, 30–45 and 45–60, respectively.

Notable differences included higher *Clostridium* sensu stricto 18 in CK (days 15–60) versus greater *Clostridium* sensu stricto 12 in JP. JP treatment also showed elevated minor LAB (*Weissella*, *Lactobacillus paracasei*, *Pediococcus*) and better-preserved LAB communities compared to CK (Supplementary Figures S2–S4). The stable Clostridia community (*Garciella*, *Anaerotruncus*, *Anaerosalibacter*, *Lachnospiraceae*) in JP versus declining CK populations suggested earlier silage maturation in JP-treated samples.

### 3.5 Co-occurrence patterns of bacterial community in JP-added alfalfa silage

**Figure 4:**
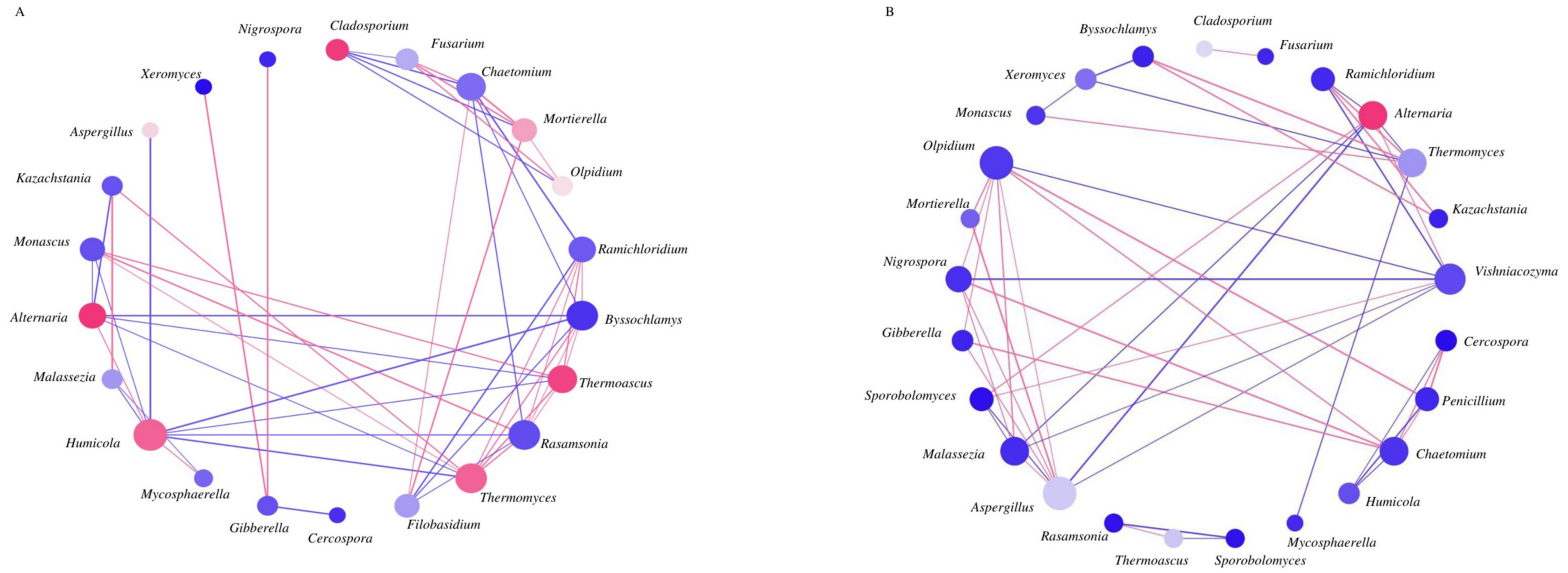
Co-occurrence network analysis among fungi OTUs (species) with relative abundances >1% in alfalfa silage for 60 days of fermentation. Bacterial co-occurrence networks (Spearman correlation, the most abundant 25 species, *p* value < 0.05, correlation>0.5) of control **(A)**, alfalfa with 4% of jujube powder **(B)**. The node represents bacterial species, node color represents bacterial abundance, and node size represents the degree. Edges are colored according to positive (red) and negative (green) correlations.

Network analysis of the top 25 OTUs at genus level in bacteria and 27 OTUS in fungi at genus level revealed distinct co-occurrence patterns between JP-treated and CK silages (Figures 3, 4 and Supplementary Tables S5–S8). In the bacterial neworks, the JP group displayed a higher number of correlation edges (143 in total, 90 positive, 53 negative) compared to (CK 109 edges, 58 positive, 41 negative) (Supplementary Tables S5, S6). The network structures differed markedly between two treatments, stronger connections observed among Clostridia (*Garciella*, *Anaerotruncus*, *Anaerosalibacter*) and *Bacillus* in CK, whereas in the JP group, edges were frequent among LAB species (*Lactobacillus*, *Leuconostoc*) and specific Clostridia (*Clostridium* sensu stricto 12, *Garciella*, *Proteus mirabilis*). While Minor populations like *Weissella* and *Pediococcus* showed minimal network impact despite their functional importance. By contrast, *Enterococcus* in both groups, and *Lactobacillus* together with *Lactococcus* in the JP group, exhibited high degree centrality and therefore exerted a stronger influence on overall network structure.

In fungi networks, the JP group displayed 47 correlation edges (27 positive, 20 negative) slightly fewer than the 50 edges observed in the control (CK) group (26 positive, 24 negative) (Supplementary Tables S7, S8). The two treatments exhibited obviously different co-occurrence structures. In CK, stronger correlations were observed among taxa such *Humicola*, *Chaetomium*, *Byssochlamys*, *Rasamsonia*, *Filobasidium*, *Thermoasc*us and *Thermomyces*. In contrast, the JP group showed increased connectivity among *Aspergillus*, *Olpidium*, *Alternaria*, *Malassezia*, and *Thermomyces*. While, the overall dominant genus (*Alternaria*) remained similar in total abundance between CK and JP, the relative abundance of certain minor taxa shifted noticeably. For instance, *Fusarium*, which showed moderate abundance in CK, appeared as a minor population in JP. More importantly, functionally relevant taxa such as *Humicola*, which exhibited high network influence in CK, were substantially reduced in JP and other fungi like *Humicola* decreased in JP. Overall, taxa with high network degree—indicating greater structural influence—included *Alternaria* in both groups, and *Humicola*, *Cladosporium*, *Thermomyces* and *Thermoascus*, which were uniquely influential in the CK network.

### 3.6 Relationships between chemical characteristics and composition of bacterial community in alfalfa silage

**Figure 5:**
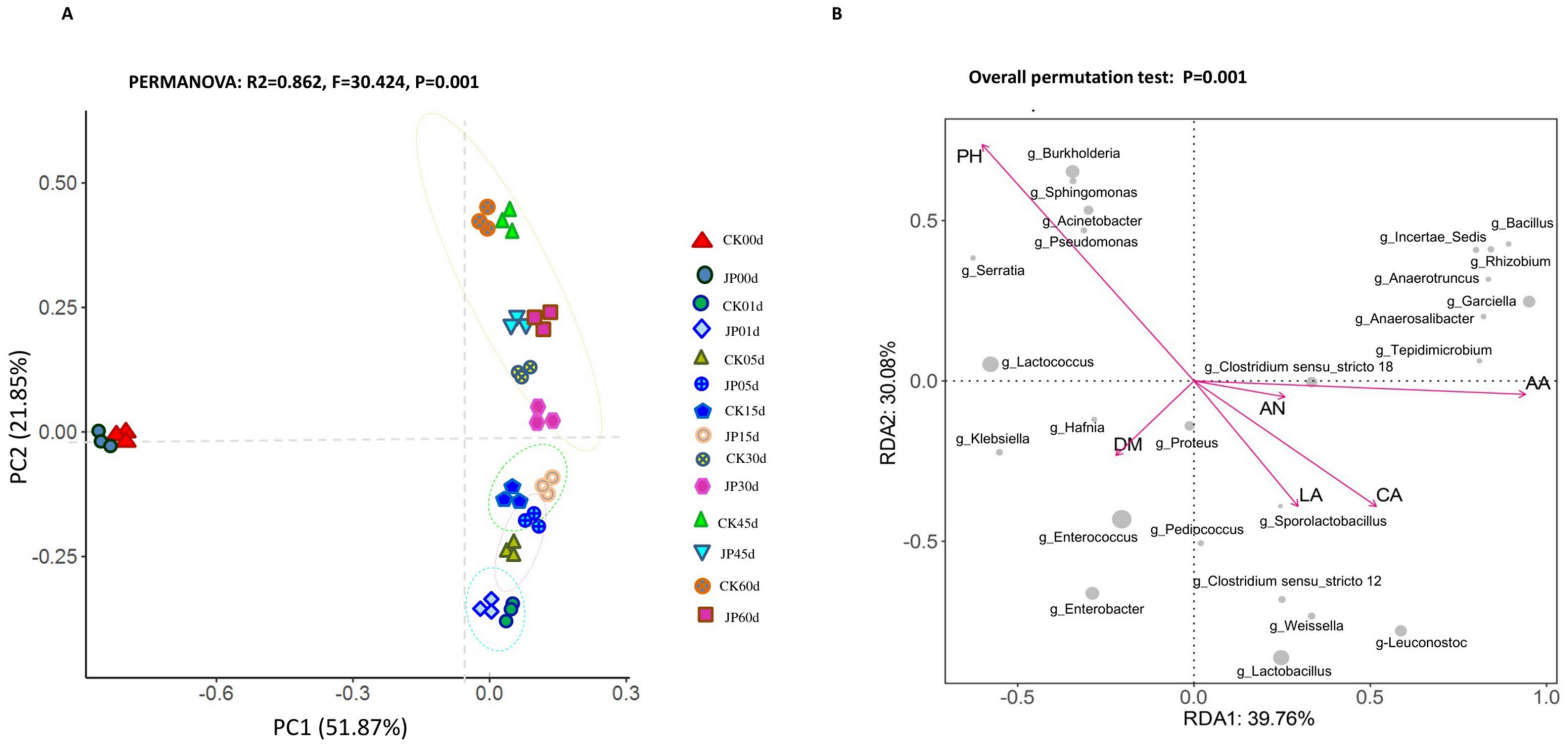
Differences in bacterial communities in alfalfa ensiling process revealed by principal component analysis based on the Bray–Curtis distances **(A)** and relationships among the top 23 OTUs in alfalfa silages and the fermentation characteristics estimated by redundancy analysis **(B)**. The explanatory (e.g., pH, lactic acid content) variables represented as arrows and silage samples as circles. CK: Control; JP: alfalfa with 4% of jujube powder; LA, lactic acid; AA, acetic acid; AN, ammonia nitrogen; DM, dry matter; CA, crude ash.

The correlation between fermentation parameters and the top 23 OTUs in alfalfa silage were analyzed through multivariate approaches. Principal coordinates analysis revealed clear separation between the fresh materials (cluster 1) and the fermented samples along the *X*-axis (Figure 5A), reflecting the rapid microbial changes during ensiling. Within the fermented samples, JP-treated and CK groups were distinguished from the in all the sampling time points, demonstrating the influence of JP addition on microbial community in ensiling. However, samples of JP treatment and CK on the same timepoint always showed closer relationships along the *Y*-axis, while the samples from early-stage (days 1–15) and late-stage (days 30–60) formed two clusters, respectively, demonstrating predictable microbial succession during fermentation. Notably, JP-treated samples reached stable community composition by day 45, while CK samples continued community changes until day 60, suggesting JP accelerated silage maturation.

LEfSe analysis identified 15 OTUs presenting differential abundances in JP treatment and CK (LDA > 2.4, *p* < 0.05), which may explain JP’s effects on microbial community. JP addition enriched *Lactobacillus* (LDA = 4.58), *Weissella*, and Enterobacteriaceae members, while suppressed *Clostridium* sensu stricto 18 (LDA = −4.65) and *Anaerotruncus* (LDA = −4.14) (Supplementary Figure S4). These shifts correlated with improved fermentation metrics, particularly reduced pH and ammonia levels. Spearman correlation analysis revealed pH and acetic acid as the strongest influencers on microbial community structure (Supplementary Figure S5 and Supplementary Tables S9–S11). JP-treated silage showed negative correlations with pH throughout the fermentation (*p* = 0.001), indicating its superior acidification (Supplementary Figure S5).

**Figure 6:**
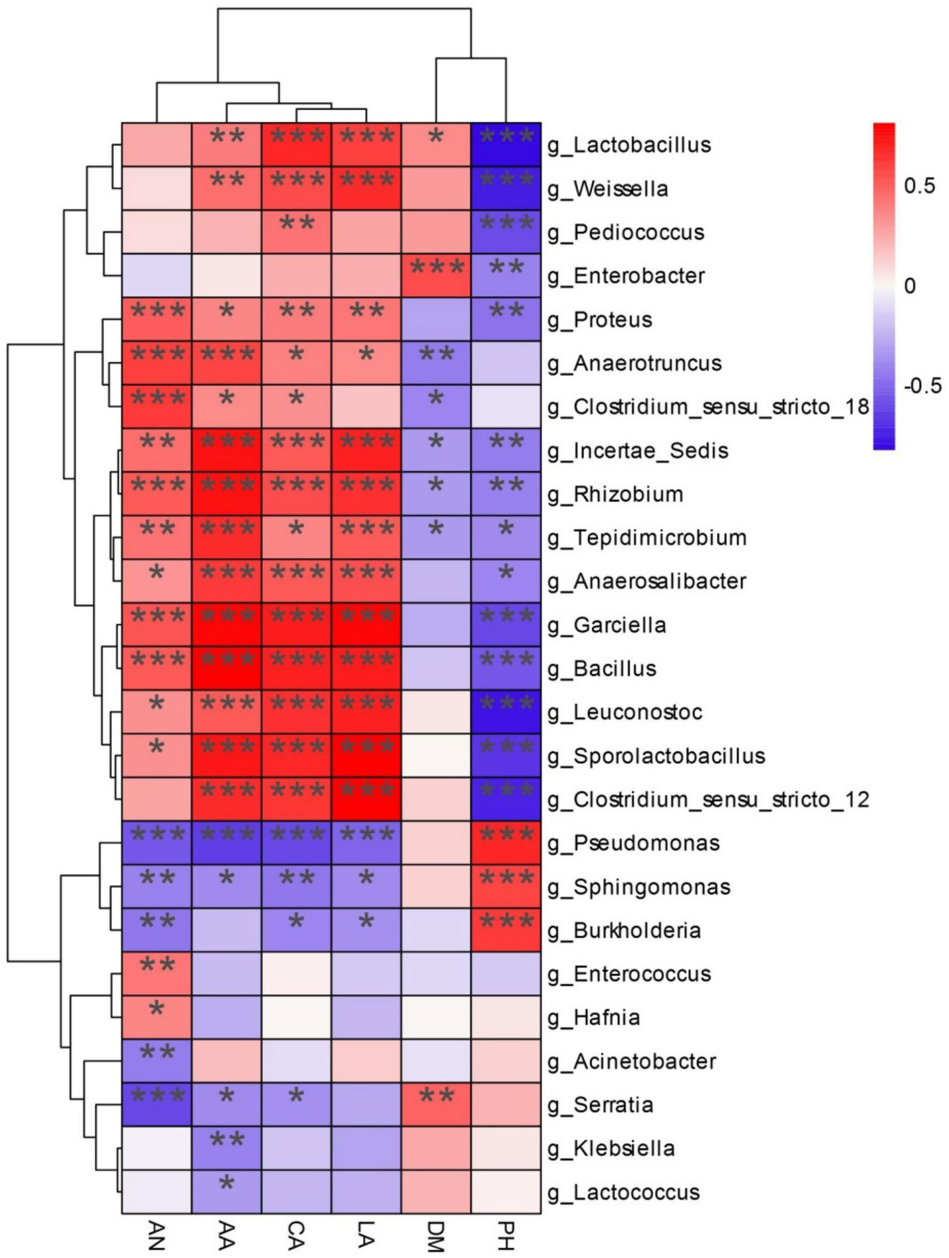
Spearman correlation heatmap of abundance of the top 23 abundant bacterial OTUs and fermentation properties in alfalfa silage for 60 days. LA, lactic acid; CA, crude ash; AA, acetic acid; AN, ammonia nitrogen; DM, dry matter; and pH. ^*^p < 0.05, ^**^p < 0.01, and ^***^p < 0.001.

Detailed correlation patterns emerged between specific OTUs and fermentation parameters are shown in Figures 5B, 6. Taxa of Non-LAB (*Burkholderia*, *Sphingomonas*) positively correlated with pH but negatively with production of acids during ensiling, while LAB showed inverse relationships. The JP-upregulated OTUs (*Enterobacter*, *Serratia*, *Lactococcuss*) were positively associated with dry matter but negatively with ammonia-N; while the downregulated taxa (*Proteus mirabilis*, *Bacillus-Clostridium*) showed opposite patterns. The OUT *Pediococcus* was uniquely positive correlated with crud ash content in JP-treated silage, suggesting that microbes may contribute to the mineral retention.

### 3.7 Analysis of functional potential in the fungal and bacterial communities

**Figure 7:**
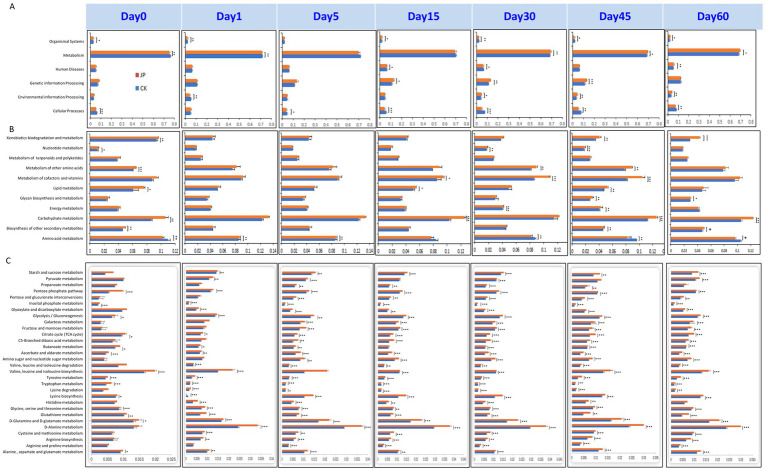
Bacterial potential metabolic pathways of alfalfa silage (CK) and with 4% of jujube powder (JP) sampled at different growth stages at pathway level 1 **(A)**, level 2 **(B)**, level 3 **(C)**.

The KEGG functionalities of bacterial communities during alfalfa ensiling, with or without jujube powder (JP), revealed that pathways categorized under “Metabolism” were dominated at first level (Figure 7A and Supplementary Table S12). At level 2, “Amino acid metabolism” and “Carbohydrate metabolism” were the most prominent (Figure 7B and Supplementary Table S13). Compared to the control (CK), JP-treated silage exhibited consistently higher relative abundances (*p* < 0.05) of “Carbohydrate metabolism” and “Metabolism of other amino acids” across all fermentation time points. Additionally, pathways including “Metabolism of cofactors and vitamins,” “Amino acid metabolism,” and “Nucleotide metabolism” were significantly elevated on day 0, 1, 5, 15 and 60.

At level 3 (Figure 7C and Supplementary Table S14), bacterial communities in JP showed significantly higher abundances (*p* < 0.05) of “Valine, leucine and isoleucine biosynthesis” throughout the ensiling process. Multiple carbohydrate and amino acid metabolic pathways were also enriched across all sampling days, including “D-Alanine metabolism,” “D-glutamate metabolism Glutathione metabolism,” “Galactose metabolism,” “Starch and sucrose metabolism,” “Glycolysis/Gluconeogenesis,” “Pyruvate metabolism,” and “Pentose phosphate pathway.” Several other pathways—such as “Cysteine and methionine metabolism,” “Alanine, aspartate and glutamate metabolism,” “Glycine, serin and threonine metabolism,” “Lysine biosynthesis,” “Glyoxylate and dicarboxylate metabolism” and “Histidine metabolism,” were significantly enriched on day 0 and 5. Notably, key acidogenic pathways—including “Glycolysis / Gluconeogenesis,” “Pentose phosphate pathway,” and “D-Alanine metabolism”—were consistently higher in JP than in CK throughout the fermentation period.

**Figure 8:**
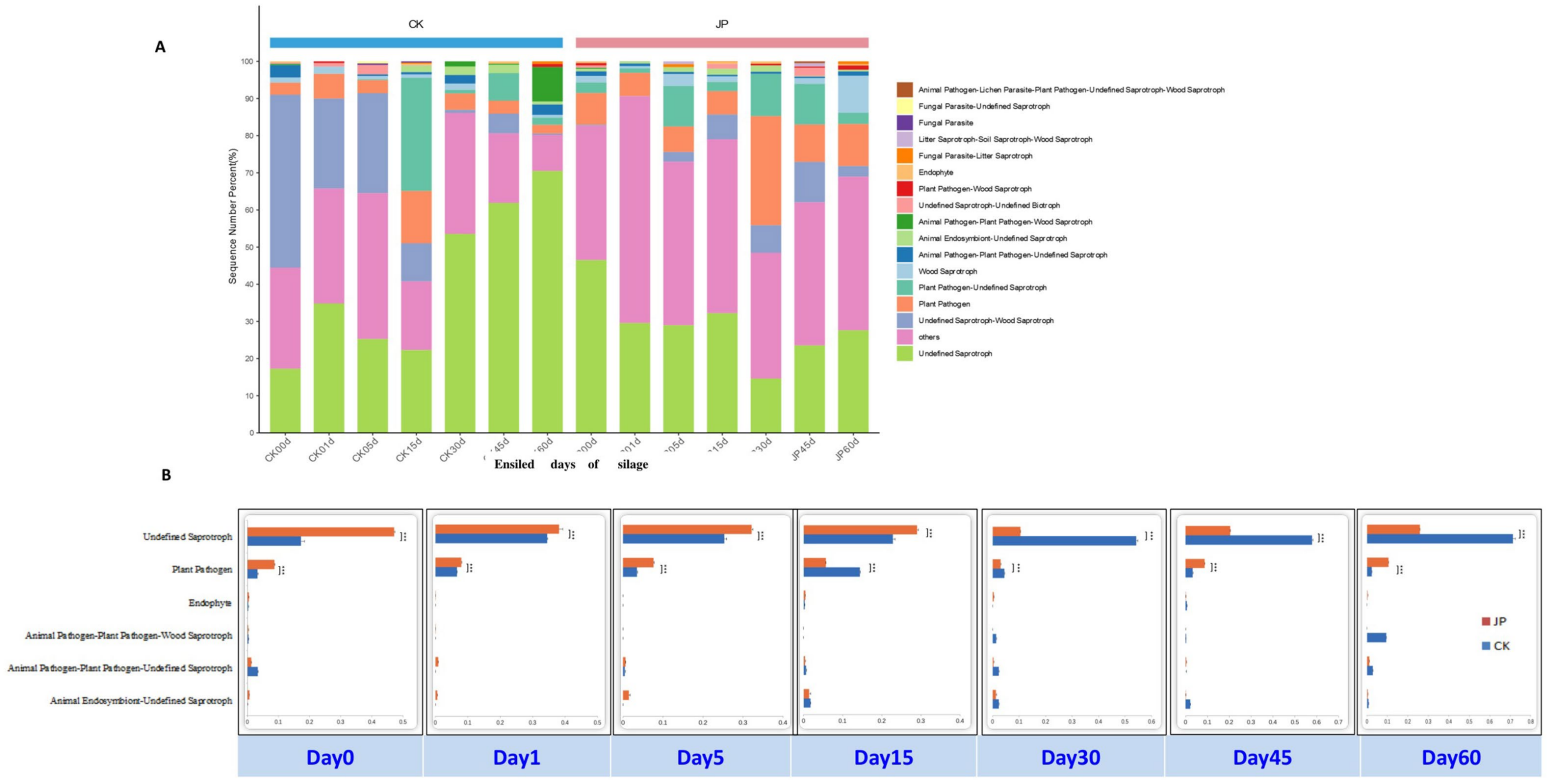
The bar plots showing the variations in composition of fungal functional group inferred by FUNGuild **(A)** and their statistical differences in alfalfa silage and with 4% of jujube powder for 0, 1, 5, 15, 30, 45, 60 days **(B)**. ^*^0.01 < *p* ≤ 0.05, ^**^0.001 < *p* ≤ 0.01, and ^***^p ≤ 0.001. CK, alfalfa silage; JP, with 4% of jujube powder silage.

Fungal functional composition, predicted by FUNGuild, is presented in Figure 8A (Supplementary Table S15). In CK, the initial community was dominated by “Undefined Saprotroph-Wood Saprotroph” (46.7%), followed by “Undefined Saprotroph” (17.5.6%) and “Plant Pathogen” (3.03%). During ensiling, “undefined Saprotroph” group increased from day 0 to day 1, then declined until day 30, before rising again toward day 60. In JP, this functional group accounted for 47.6% initially but declined steadily throughout fermentation, falling to 10% by day 60—a level lower than that in CK. “Plant Pathogen” remained at low abundances (<4%) in CK, but increased over time, stabilizing around 3% by the end, with only slight rises days 45 and 60. Meanwhile, “Undefined Saprotroph-Wood Saprotroph,” which dominated CK initially (46.7%), maintained low relative abundance in JP throughout the ensiling process.

## 4 Discussion

### 4.1 Effects of jujube powder addition on high moisture alfalfa silage quality

Jujube powder (JP), a widely used feed additive, was employed by this study to address issues in alfalfa silage, such as inadequate fermentation and susceptibility to spoilage caused by insufficient water-soluble carbohydrates (WSC) and high buffering capacity. The pH value, content of dry matter, and water-soluble carbohydrate concentration in fresh alfalfa, and absence of organic acids or NH_3_-N in both the raw materials (Table 1) were consistent with the previous reports (Yang et al., 2022).

By the end of ensiling, comparing with CK, the silage quality characters, pH values, LA content, NH_3−_N content (Table 2) and the microbial community composition (Figures1, 2) or diversity (Supplementary Figure S1) in JP treated ensiling reached to stable 15 days earlier, demonstrating that the JP addition accelerated the ensiling procedure. Although the silages of both the treatments fitted the thresholds for well-preserved silage (NH_3_-N < 10% of total nitrogen; pH < 4.5) (Kung et al., 1998, 2018) by the end of ensiling, the fermentation procedure in JP-added ensiling was more effective for degreasing pH and production of LA, while inhibiting plant enzymatic activity (NH_3_ release by proteolysis) (Ding et al., 2013; Tao et al., 2012). The temporary pH increase occurring on day 5 in both groups could be due to reduced organic acid production and NH_3_-N release from protein degradation (Ding et al., 2013; Tao et al., 2012). In the final silage (60 days of fermentation), the greater contents of DM and lactic acid, and the lower pH value, NH_3_-N levels, acetic acid content and propionic acid content in JP added silage than that in CK (Figure 1) were consistent with the previous results (Ding et al., 2013; Liu et al., 2016; Rajabi et al., 2017; Tian et al., 2017; Weiss and Underwood, 2009) and clear evidenced that the JP addition improved the quality of high moisture silage. In our study, all the important quality features (Figure 1) reached the values comparable to those achieved in pre-wilted alfalfa silage (Zhang et al., 2018), implying that 4% (w/w) JP addition can be used to replace the energy- and labor-intensive pre-wilting in alfalfa ensiling.

### 4.2 Influences of jujube powder addition on high moisture alfalfa silage quality by regulating microbial community

Although the compensative physiochemical/microbial traits of jujube powder (JP) in fresh alfalfa (Table 1) provided a basis for improving alfalfa silage quality, the underlying microbiological mechanisms and how these traits modify the fermentation process remained unclear. Therefore, we further investigated the fermentation and microbial community dynamics during ensiling.

First, the higher water-soluble carbohydrate (WSC) and dry matter (DM) contents, together with the greater initial lactic acid bacteria (LAB) counts in JP (Table 1), promoted rapid oxygen consumption by the aerobic-facultative anaerobic bacteria (*Enterobacteria*, *Garciella*, *Acinetobacter*, and *Burkholderia* etc.). This in turn stimulated the subsequent growth of anaerobic LAB (Figure 2). Resulting in substantial lactic acid production and a sharp decline in pH during the early stages (day 1) of ensiling (Table 2), consistent with typical fermentation patterns (Muck et al., 2018).

Second, JP treatment significantly modified bacterial community composition compared with CK. Throughout ensiling, JP silage contained fewer *Enterococcus* and *Leuconostoc* but more *Lactobacillus*, likely related to the changes in DM and LAB content induced by JP addition (Su et al., 2023). Notably, during days 15–60, JP-supplied silage showed lower abundance of *Clostridium* sensus stricto 18 and higher abundances of *Clostridium* sensus stricto 12, possibly due to fructose-enhanced *Lactobacillus* growth (Endo et al., 2018). Increased abundances of minor LAB genera such as *Weissella*, *Sporolactobacillus* and *Pediococcus* in JP silage (Supplementary Figures S2, S4) may have further contributed to quality improvement. The persistence of *Enterococcus* in CK implied incomplete fermentation (Liu et al., 2022), while its stabilization in JP indicated earlier silage maturation.

In both treatments, rapid microbial succession occurred during the early ensiling, with *Burkholderia* and *Acinetobacter* disappearing while *Lactococcus*, *Enterococcus* and *Enterobacter* increased markedly by day 1. This shift was likely driven by the large *Lactococcus* that rapidly initiated fermentation. Bacterial community analysis identified two ensiling phases: early (1–15 days) and middle-late (30–60 days). JP treatment promoted earlier maturation, as evidenced by tight clustering of samples from days 45–60, whereas CK samples remained dispersed throughout fermentation. These patterns align with previous reports on alfalfa silage microbial dynamics (Liu et al., 2019; Zhao et al., 2021).

JP addition also alters fungal community composition, potentially influencing silage quality through mechanisms described in previous studies (Cheli et al., 2013; Wang et al., 2023). JP addition suppressed several potentially harmful fungi, including *Phaeosphaeria*, *Aspergillus*, *Penicillium* and *Fusarium*, particularly, *Alternaria* population were decreased by the ensiling process, demonstrating its antifungal properties during ensiling, which might be related to the increased population and metabolic activities of LAB (Fugaban et al., 2023). *Alternaria*, *Aspergillus*, *Penicillium* and *Fusarium* are well known mycotoxin producers (Kushwaha et al., 2025), while *Phaeosphaeria* is a genus with many phytopathogens and bioactive compounds (El-Demerdash, 2018). This change in fungal composition further supports that JP addition could improve the quality of alfalfa silage by reducing the mycotoxin levels (Muck et al., 2018).

Third, JP supplementation regulated the microbial community structure. Unlike that in sorghum silage (Gallagher et al., 2018), JP-amended alfalfa silage showed an apparent decoupling between LAB community composition and total abundance, especially in later stages. This suggests that the composition of LAB may exert a stronger influence on fermentation outcomes than overall abundance. Specifically, homofermentative species such as *Lactobacillus plantarum* dominated early fermentation when sugars are abundant (Li et al., 2018; Wang et al., 2017), indicating significant microbial succession throughout the ensiling process. This pattern aligns with previous observations that only specialized species like *Lactobacillus buchneri* remain active at low densities during late fermentation stages (Driehuis et al., 1999). JP treatment significantly altered this succession by reducing the abundance of *Enterococcus* and *Leuconostoc* while increasing *Lactobacillus* populations-a shift associated with improved fermentation outcomes (Su et al., 2023). Both JP addition and ensiling duration enhanced microbial diversity and richness, contrary with early findings that LAB and molasses addition in soybean silage decreased bacterial diversity (Ni et al., 2017).

### 4.3 Effects of jujube powder addition on bacterial and fungal co-occurrence networks and microbial interactions

Network analysis revealed that JP restructured microbial interaction, strengthening association among beneficial lactic acid bacteria (LAB) while weakening undesirable connections involving Clostridia. Contrary to previous reports of higher bacterial diversity in untreated silage (Ni et al., 2017), our results showed that JP simplify microbial interactions while promoting beneficial microbial relationships—consistent with findings from LAB-inoculated silages (Liu et al., 2022) and other additive-treated systems (Bai et al., 2021). This reduced network complexity was associated with improved fermentation quality, in agreement with previous studies (Bai et al., 2021).

Certain bacterial taxa played central roles in shaping the network structure. In the control (CK), *Enterococcus* significantly contributed to network connectivity. Although taxonomically and physiologically classified as a lactic acid bacterium, *Enterococcus* can contribute to early acidification; however, in silage fermentation practice, it is generally not regarded as a beneficial or efficient probiotic lactic acid bacterium due to its slow acid production rate, limited acid yield, and the propensity of some of species to produce ammino N (Muck et al., 2018). The persistence of *Enterococcus* in CK may therefore be associated with elevated amino-N levels and accelerated silage deterioration in the mid to late stages of ensiling. These undesirable characteristics may further explain the stronger co-occurrence observed between *Enterococcus* and know spoilage-associated taxa as Clostridia (e.g., G*arciella*, *Anaerosalibacter*) and *Bacillus*—all of which are recognized contributors to ammonia production and undesirable fermentation (Borreani et al., 2018).

In contrast, JP treatment reshaped the bacterial network, with Enterococcus, Lactobacillus, and Lactococcus emerging as key structural taxa. This restructuring favored positive interactions among LAB species (e.g., *Lactobacillus*, *Pediococcus*) and specific Clostridia (*Clostridium* sensu stricto 12, *Proteus mirabilis*), which correlated with higher lactic acid accumulation and better pathogen control. Simultaneously, JP suppressed potentially harmful genera such a *Garciella*, *Anaerotruncus* and *Anaerosalibacter* (Supplementary Figure S3). The absence of butyric acid in both treatments corresponed to low clostridial abundance, contrasting with the report of strong clostridial activity in high-moisture alfalfa silage (Zheng et al., 2017). Furthermore, JP treatment reduced undesirable bacteria including *Anaerosalibacter*, *Garciella*, and *Enterobacter*—known to promote ammonia and butyric acid formation (Borreani et al., 2018; Lawson et al., 2004; Pahlow et al., 2003; Togo et al., 2019; Zhang et al., 2018). Compared with commercial LAB inoculants and other chemical additives (Ding et al., 2013; Ni et al., 2017), JP proved to be a more supported greater bacterial diversity, which may contribute to the improved silage quality.

Successional dynamics of three core LAB genera were distinct in JP-treated silage. *Lactococcus* upregulated by JP, rapidly initiated acid production, peaking on day1. This was early (day 1) subsequent fermentation stages showed characteristic successional patterns, with *Lactococcus* followed by followed by sequential increases in *Enterococcus* (day 5), *Lactobacillus* (days 30–45) and finally *Anaerosalibacter* (days 45–60). This pattern resembles succession reported corn silage, where early dominance of *Lactococcus* is succeeded by *Lactobacillus* and *Pediococcus* species (Wang et al., 2017). In contrast, down-regulated taxa such as *Proteus mirabilis* and members of the *Bacillus-Clostridium* group showed opposing trends, consistent with their roles in ammonification (Muck et al., 2018). JP treatment altered their co-occurrence patterns, generating negative correlations with *Sphingomonas* and positive links with *Lactococcus* and *Pediococcus*.

All these findings collectively demonstrate that jujube powder acts as a multifunctional silage additive that shift microbial community structure by establishing a cooperative consortium of *Lactococcus*, *Lactobacillus* and *Pediococcus*. These three genera likely play complementary roles: *Lactococcus* drives fast-start fermentation, *Lactobacillus* sustains strong homofermentative acid production, and *Pediococcus* maintains activity under prolonged acidic conditions. Together, they contribute to a more efficient and stable ensiling process.

### 4.4 Effects of jujube powder addition on fungal and bacterial functional profiles in relation to silages quality

The predominant bacterial functional pathways at level 2 in both CK and JP treatments were associated with carbohydrate and amino acid metabolism. This aligns with expectations, as anaerobic ensiling relies on the conversion of water-soluble carbohydrates (WSC) into organic acids—primarily lactic acid—by lactic acid bacteria (LAB) under oxygen-limited conditions (McDonald et al., 1991). Furthermore, the ensiling process in the JP treatment effectively suppressed amino acid metabolism in many undesirable bacteria, resulting in an overall reduction of this activity compared to CK. The enhanced activities of pathways such as “D-Alanine metabolism,” “Valine, leucine and isoleucine biosynthesis” and glycolysis/gluconeogenesis in the JP group were primarily linked to the rapid proliferation of LAB. This contributed to maintaining protein stability by limiting proteolysis.

D-alanine is not primarily a metabolic energy substrate; rather, it serves as an essential structural component for peptidoglycan biosynthesis in the cell walls of lactic acid bacteria (LAB) (Steen et al., 2005; Palumbo et al., 2004). This role support cell wall integrity, ensuring the survival and functional dominance of these key fermentative bacteria in the acidic, anaerobic silage environment (Palumbo et al., 2004). The presence of D-amino acids, produced by microorganisms during fermentation, has been associated with enhanced taste profiles (e.g., umami, sweet) in foods like cheese and vinegar (Kanauchi and Matsumoto, 2023). In silage, acetic acid derived from metabolism pathways involving alanine or pyruvate acts as an effective antifungal agent. An appropriate concentration of acetic acid significantly improves the aerobic stability of silage after opening, thereby helping to prevent spoilage.

In contrast to D-alanine, valine is a key indicator and participant in detrimental fermentation processes, notably the “Stickland fermentation” (Muck et al., 2018). This process represents the most harmful function of valine in silage, leading to the production of butyric acid and ammonia, along with substantial loss of true protein, which reduces the feed’s nutritional value. Moreover, during later ensiling stages or after exposure to air, certain microbes can maetabolize valine into compounds like isobutanol, imparting an undesirable alcoholic odor.

The higher NH₃-N content observed in CK compared to JP can thus be attributed to divergent roles of D-alanine metabolism and valine metabolism. Concurrently, the increased glycolytic activity detected in the JP treatment indicates an enhanced capacity of LAB to utilize carbohydrates, promoting efficient conversion of glucose to lactic acid by homofermentative strains (Giacon et al., 2022). This more efficient fermentation contributed to the improved preservation outcomes observed with jujube powder addition.

Fungal ecological functions were analyzed using the FUNGuild tool (Nguyen et al., 2016). The analysis revealed a consistently higher proportion of the functional guild “Undefined Saprotroph-Wood Saprotroph” in the control (CK) than in the JP treatment throughout fermentation. This indicates an elevated risk of saprotrophic (decay) activity in untreated alfalfa silage. Furthermore, an increase in the proportion of certain fungal species associated with animal pathogens was noted on days 45 and 60, suggesting prolonged fermentation may heighten this risk. The JP treatment effectively suppressed “Undefined Saprotroph” fungi. This suppression likely resulted from the rapid and dominant fermentation initiated by LAB in the JP-treated silage, which inhibited fungal populations through acid production and competitive exclusion.

## 5 Conclusion

Supplementation with jujube powder (JP) significantly shortened the required ensiling duration and optimized key fermentation parameters (pH, lactic acid, volatile fatty acids, and NH_3_-N) in high-moisture alfalfa, achieved fermentation quality comparable to traditional wilted silage. A novel microbial consortium primarily composed of *Lactobacillus*, *Pediococcus* and *Lactococcus* was identified and shown to have a strong positive correlation with improved silage quality. Within this consortium, *Lactobacillus* (homofermentative, strong acid producer), *Pediococcus* (homofermentative, acid-tolerant), and *Lactococcus* (rapid fermentation starter) are likely to play complementary roles across different fermentation stages. These findings suggest that JP addition to fresh alfalfa can effectively replace the energy- and labor-intensive pre-wilting step. Furthermore, the identified microbial consortium provides a promising candidate for the future development of high-efficiency silage inoculants.

## Data Availability

The datasets presented in this study can be found in online repositories. The names of the repository/repositories and accession number(s) can be found in the article/Supplementary material.

## Footnotes

1
https://www.chinabaogao.com/market/202412/737909.html
