# Supplementary material for: Jujube powder supplementation optimized high-moisture alfalfa silage through regulating microbial community
Source: Front Microbiol. 2026 Jan 12;16:1740083. doi: 10.3389/fmicb.2025.1740083 (PMC12833439; doi:10.3389/fmicb.2025.1740083)
Supplement: Supplementary file 1 [file Presentation_1.PPTX]

## Slide 1
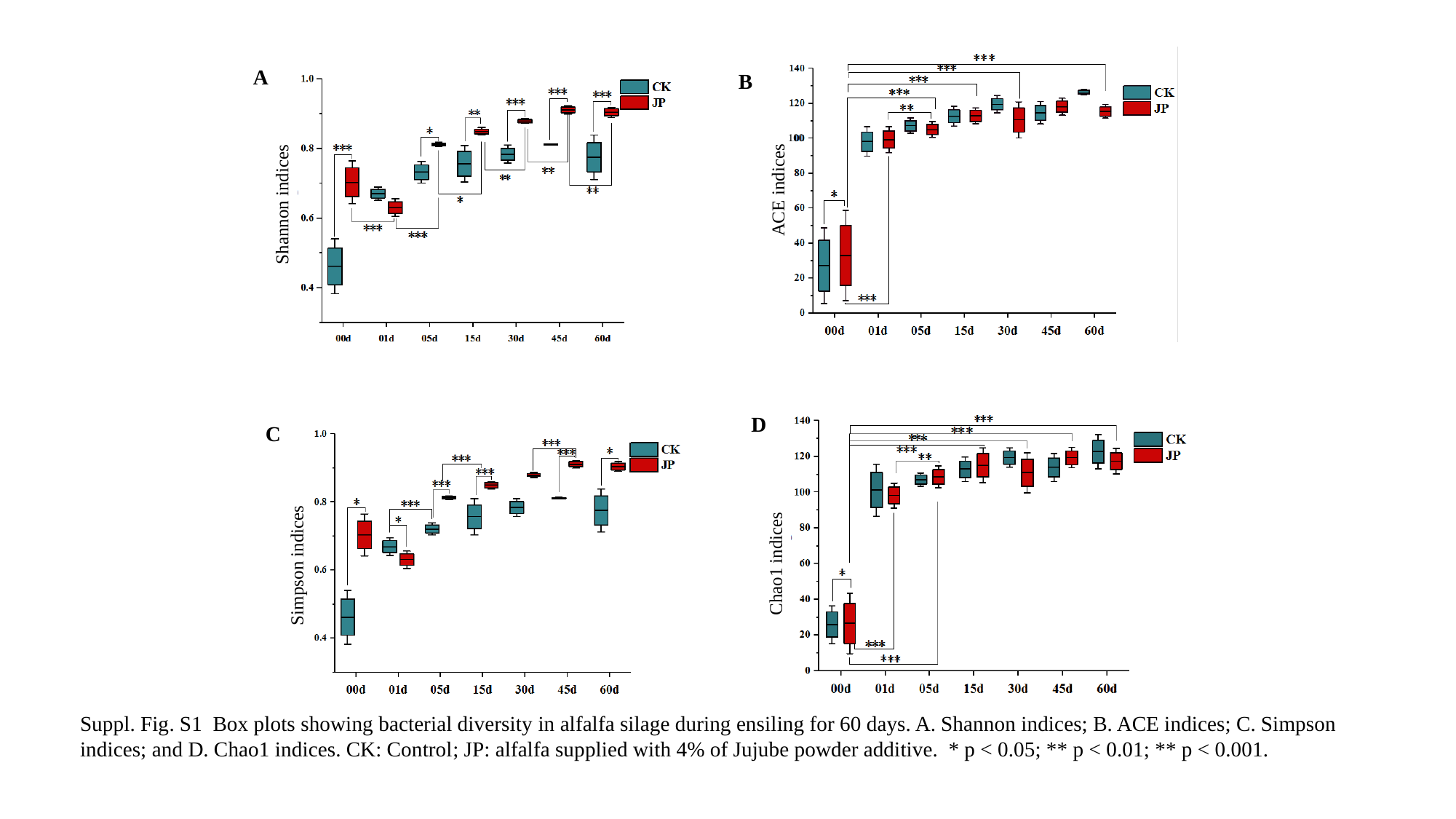

A
B
B
100
ACE indices
Shannon indices
D
C
Chao1 indices
Simpson indices
Suppl. Fig. S1 Box plots showing bacterial diversity in alfalfa silage during ensiling for 60 days. A. Shannon indices; B. ACE indices; C. Simpson indices; and D. Chao1 indices. CK: Control; JP: alfalfa supplied with 4% of Jujube powder additive. * p < 0.05; ** p < 0.01; ** p < 0.001.

## Slide 2
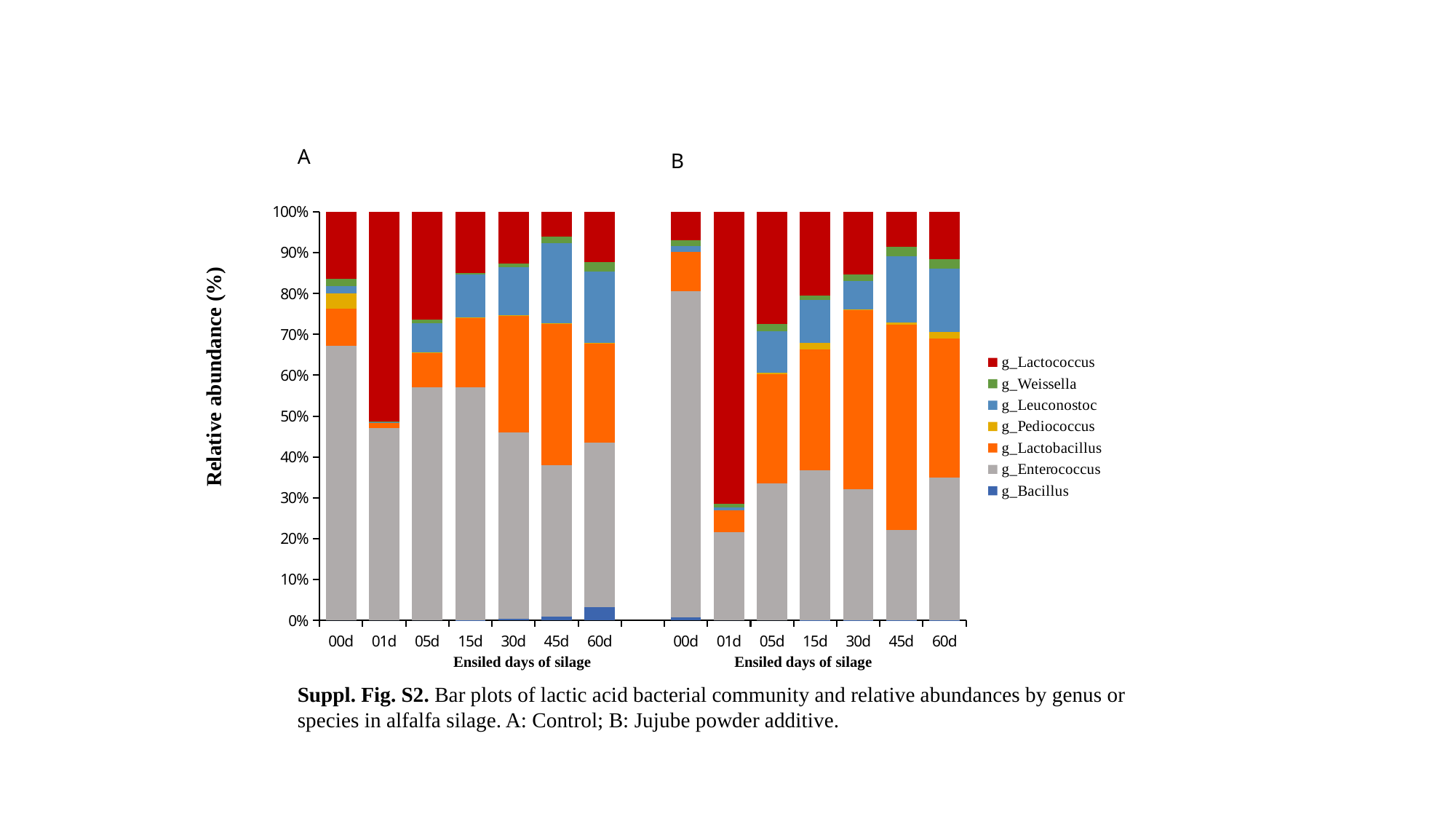

A
B
### Chart
| Category | g_Bacillus | g_Enterococcus | g_Lactobacillus | g_Pediococcus | g_Leuconostoc | g_Weissella | g_Lactococcus |
|---|---|---|---|---|---|---|---|
| 00d | 0.0 | 12.333333333333334 | 1.6666666666666667 | 0.6666666666666666 | 0.3333333333333333 | 0.3333333333333333 | 3.0 |
| 01d | 0.0 | 17306.0 | 452.3333333333333 | 0.0 | 75.66666666666667 | 63.0 | 18831.666666666668 |
| 05d | 0.0 | 29447.666666666668 | 4303.333333333333 | 109.0 | 3697.6666666666665 | 465.0 | 13608.0 |
| 15d | 7.666666666666667 | 36154.0 | 10750.666666666666 | 83.33333333333333 | 6634.0 | 282.0 | 9447.666666666666 |
| 30d | 103.66666666666667 | 13918.0 | 8739.333333333334 | 44.333333333333336 | 3585.0 | 272.0 | 3842.0 |
| 45d | 217.0 | 8724.0 | 8229.333333333334 | 6.666666666666667 | 4583.333333333333 | 398.0 | 1430.6666666666667 |
| 60d | 668.3333333333334 | 8437.333333333334 | 5089.0 | 12.333333333333334 | 3647.6666666666665 | 505.3333333333333 | 2563.0 |
| | None | None | None | None | None | None | None |
| 00d | 0.3333333333333333 | 38.333333333333336 | 4.666666666666667 | 0.0 | 0.6666666666666666 | 0.6666666666666666 | 3.3333333333333335 |
| 01d | 0.0 | 5727.0 | 1438.3333333333333 | 0.0 | 194.33333333333334 | 205.0 | 18994.333333333332 |
| 05d | 0.0 | 20097.333333333332 | 16025.666666666666 | 183.33333333333334 | 6009.0 | 1135.6666666666667 | 16419.0 |
| 15d | 4.666666666666667 | 19175.333333333332 | 15421.666666666666 | 904.0 | 5466.666666666667 | 585.0 | 10667.0 |
| 30d | 14.0 | 13120.666666666666 | 17993.333333333332 | 44.333333333333336 | 2868.6666666666665 | 676.6666666666666 | 6243.333333333333 |
| 45d | 12.0 | 7930.666666666667 | 18060.0 | 152.33333333333334 | 5839.333333333333 | 848.0 | 3050.6666666666665 |
| 60d | 12.666666666666666 | 7546.333333333333 | 7348.0 | 347.0 | 3371.3333333333335 | 475.3333333333333 | 2516.6666666666665 |Relative abundance (%)
Ensiled days of silage
Ensiled days of silage
Suppl. Fig. S2. Bar plots of lactic acid bacterial community and relative abundances by genus or species in alfalfa silage. A: Control; B: Jujube powder additive.

## Slide 3
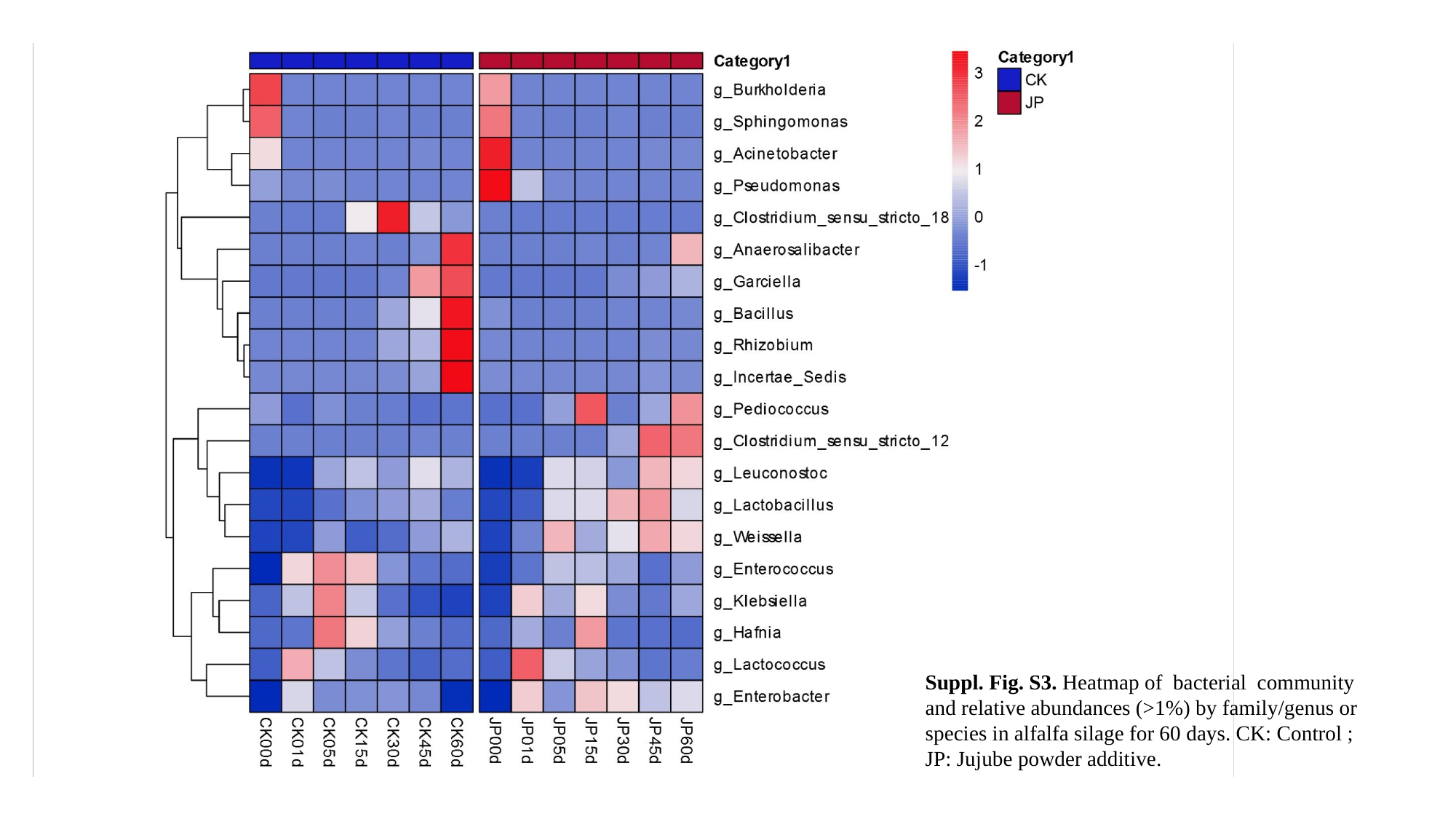

Ensiled days of silage
Suppl. Fig. S3. Heatmap of bacterial community and relative abundances (>1%) by family/genus or species in alfalfa silage for 60 days. CK: Control ; JP: Jujube powder additive.

## Slide 4
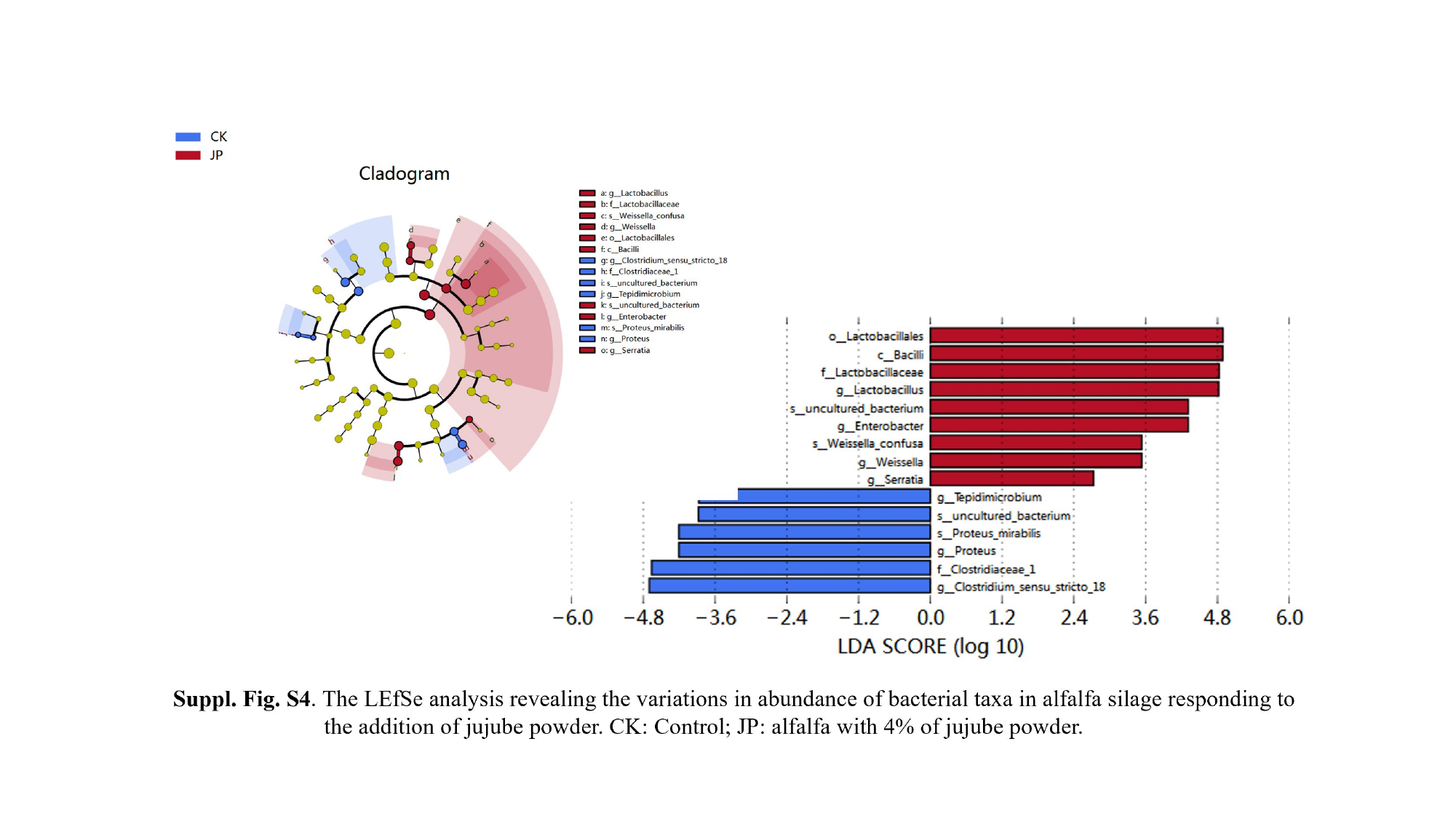

## Slide 5
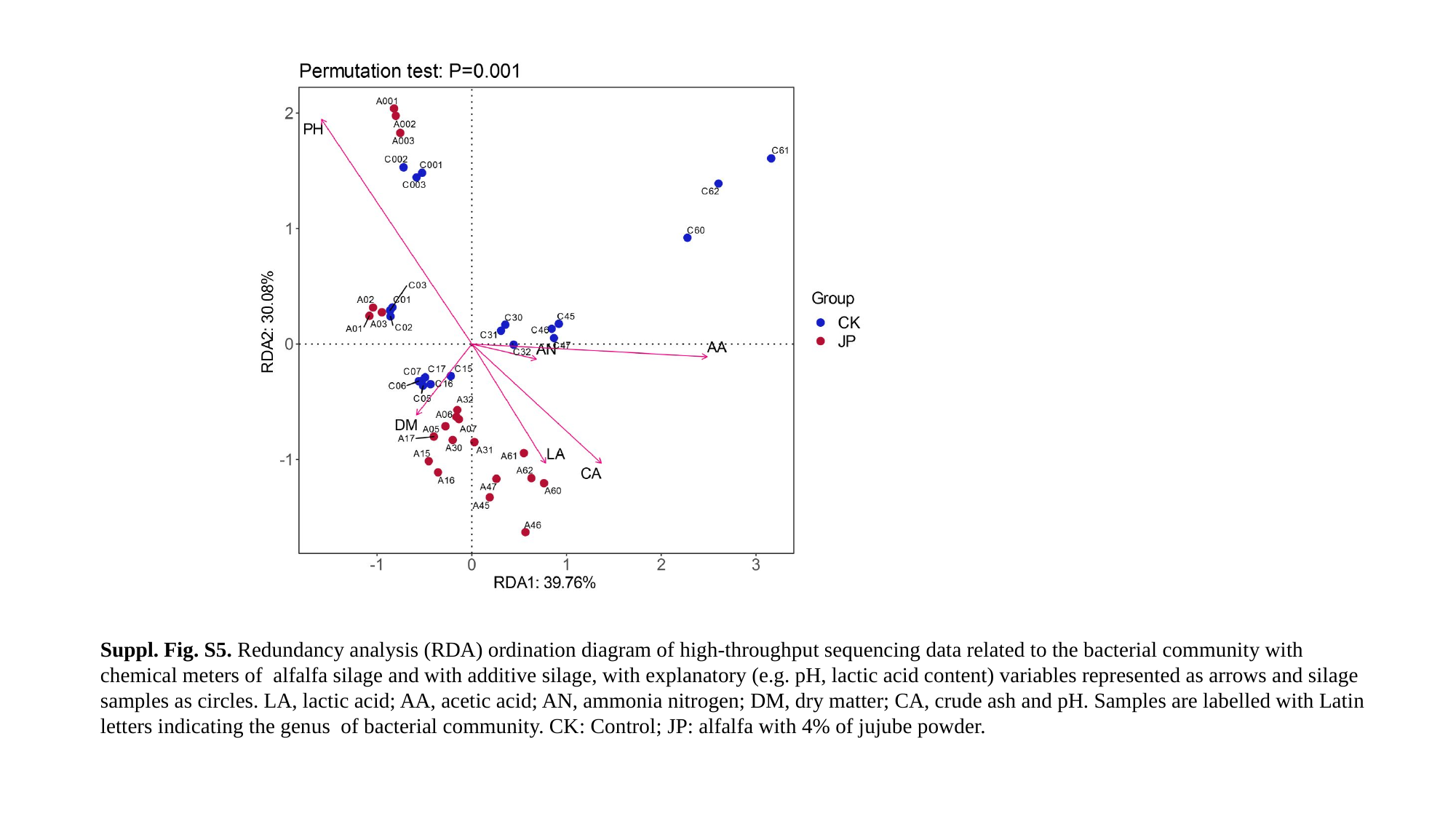

Suppl. Fig. S5. Redundancy analysis (RDA) ordination diagram of high-throughput sequencing data related to the bacterial community with chemical meters of alfalfa silage and with additive silage, with explanatory (e.g. pH, lactic acid content) variables represented as arrows and silage samples as circles. LA, lactic acid; AA, acetic acid; AN, ammonia nitrogen; DM, dry matter; CA, crude ash and pH. Samples are labelled with Latin letters indicating the genus of bacterial community. CK: Control; JP: alfalfa with 4% of jujube powder.
